# An Advance Announcement

**Published:** 1910-12-31

**Authors:** 


### "AN ADVANCE ANNOUNCEMENT."

With reference to the Annotation which appeared under this heading in The Hospital of December 17, 1910, page 337, we have received a letter from Mr. John Murray, of 50 Albemarle Street, in which he states :—

"My attention has been called to a paragraph in The Hospital for December 17 reflecting on an announcement of Dr. H. C. Ross's work on " Induced Cell Reproduction and Cancer." Allow me to inform you that Dr. Ross knew nothing whatever about the advertisement in question until after it had appeared. It was prepared in my office and I therefore hold myself responsible. The instant Dr. Ross saw the advertisement he telegraphed to me, and I at once took pains to stop it in every quarter in which it had not already appeared."

[This letter shows that Dr. H. C. Ross had no knowledge of the intention to issue the notice we referred to and that he is in complete agreement with the views we expressed. It is matter for congratulation that this is now placed beyond dispute, and we are indebted to Mr. Murray for enabling us to give publicity to the fact.—Ed. The Hospital.]

